# 

**Published:** 1892-07

**Authors:** 


### LITERARY.

### THE ST. LOUIS HYGIENIC COLLEGE OF PHYSICIANS AND SURGEONS.

The 6th ANNUAL COURSE of Instruction in this excellent and fully equipped Institution, FOR BOTH SEXES, on absolutely equal terms, will commence on the 29th of next September.

The course of study is thorough, and comprehends all branches of MEDICINE and SURGERY taught in our *Old-School* Colleges; besides Hygeio-Therapy, Sanitary Engineering and Physical Culture.

The system has met with almost unexampled success, as it ought to, for its methods are founded in Natural Law, and its remedies are aids to *Nature's Self.*

See advertisement in our columns. On the part of Men and Women alike, Hall's Journal wishes it God speed.

HOME WARMING AND VENTILATION.—This is the title of a compact little work issued by The Herenden Manufacturing Co., Geneva, N. Y., compiled mainly from the essays of distinguished authors upon a subject which so materially affects the well-being of every household. The work deals with every question which enters, even remotely, into the subject of ventilating and warming dwelling houses and public assembly rooms, and is as full of information as its ample pages can hold.

THE HANDSOME SOUVENIR OF THE VISIT OF THE AMERICAN MEDICAL ASSOCIATION TO DETROIT; issued by those extensive Manufacturing Druggists, Parke, Davis & Co., was one of the most interesting features of the occasion. The Souvenir is descriptive of the several departments of P. D. & Co's immense works, from the storage of the raw material to the shipping department; amply and truthfully illustrated from photographic views, and serves as a fit memento of the late visit of the Association.

JENNESS MILLER ILLUSTRATED MONTHLY, for July, contains many attractive features. There is a portrait and sketch of Mrs. Charles H. Parkhurst, wife of the great New York reformer. Mrs. Jenness Miller writes of "Unimportant Trifles of Women." Hon. George L. Catlin, Consul at Zurich, writes of "A First Glimpse of Switzerland." Vance Thompson picturesquely describes "Children of the Streets." Foster Coates describes "A Visit to Archdeacon Farrar." There are numbers of other articles chiefly by well-known writers of equal interest. The proprietors have undertaken to make JENNESS MILLER ILLUSTRATED MONTHLY, the great family magazine of America. Price is $1 a year, 10 cents a copy of all news agents. One of the novel features of the venture is the giving away of a $1 Union suit of woman's underwear with each $1 yearly subscription to the magazine. Address, Publishers Jenness Miller Illustrated Monthly, 114 Fifth Avenue, New York.

BABYLAND AND OUR LITTLE MEN AND WOMEN FOR JULY are full of good things. The latter has a very suggestive Fourth of July story and poem, a pretty and suggestive story for vacations for poor children, a bright little sketch of "A Little Girl Ruler"—Wilhelmena, the youngest sovereign in the world, with rhymes and jingles that boys and girls delight to read, and Babyland is not a bit behind with its pretty stories and bright bits of verse and rhyme. These dainty little magazines stand without a rival in their especial field. Price $1 and 50 cents a year; 5 cents a number. D. Lothrop Co., Publishers, Boston.

